# Understanding Spaceflight-Induced Oxidative Stress and the Critical Role of Diet and Microbiome

**DOI:** 10.3390/antiox15050534

**Published:** 2026-04-24

**Authors:** Gun Kim, Yeonje Park, Yeo Kyem Lim, Ji Won Lee, Dawon Kang, Dong Kun Lee, Jae Ho Lee, Min Seok Song, Bo Hyun Lee

**Affiliations:** 1 Department of Pharmacology, College of Medicine, Catholic Kwandong University, Gangneung 25601, Republic of Korea; 2 Department of Physiology, College of Medicine, Gyeongsang National University, Jinju 52727, Republic of Korea; 3 Department of Convergence Medical Science, Gyeongsang National University, Jinju 52727, Republic of Korea; 4 Laboratory of Veterinary Pharmacology, College of Veterinary Medicine, Research Institute for Veterinary Science, Seoul National University, Seoul 08826, Republic of Korea

**Keywords:** oxidative stress, space nutrition, space food systems, gut microbiome, redox homeostasis

## Abstract

Spaceflight exposes astronauts to multiple environmental stressors that promote oxidative stress, including ionizing radiation, microgravity, circadian rhythm disruption, and psychological stress. These factors increase the production of reactive oxygen species (ROS) and disturb redox homeostasis, potentially affecting multiple physiological systems during long-duration missions. In addition to environmental challenges, nutritional factors may further influence oxidative balance in space. Space food systems rely on long-term storage and processing, which can lead to degradation of antioxidant nutrients and alterations in dietary composition. Furthermore, spaceflight conditions may modify eating behaviors and disrupt gut microbiome composition, both of which are closely linked to host redox regulation. This review examines current knowledge on oxidative stress during spaceflight and discusses how space food systems, dietary composition, and microbiome alterations interact with spaceflight stressors to influence redox homeostasis. Potential strategies to mitigate oxidative stress are also discussed, including preservation of antioxidant nutrients, optimization of dietary composition, reduction in pro-oxidant exposures, and microbiome-targeted approaches to support astronaut health during long-duration missions.

## 1. Introduction

### 1.1. Biological Significance of ROS and the Impact of Oxidative Stress

Reactive oxygen species (ROS), such as superoxide, hydrogen peroxide, and hydroxyl radicals are inevitable byproducts of aerobic metabolism, particularly mitochondrial oxidative phosphorylation [1]. Beyond mitochondria, peroxisomes, endoplasmic reticulum, and enzymatic systems, including NADPH oxidases, xanthine oxidase, and cytochrome P450, also contribute to intracellular ROS generation [2]. At physiological levels, ROS are not merely byproducts of metabolism but also serve as essential regulators of cellular processes. Controlled ROS production acts as a second messenger in redox signaling, influencing pathways such as MAPK/ERK and PI3K-AKT-mTOR, and regulates transcription factors like NF-κB and Nrf2, which are known to be involved in cell proliferation, differentiation, and survival [3,4,5,6,7]. At the molecular level, these signaling functions are primarily mediated through the selective and reversible oxidation of redox-sensitive cysteine thiol groups in target proteins, allowing transient modulation of protein activity without irreversible oxidative damage. Balanced redox signaling under physiological conditions is often referred to as oxidative eustress, highlighting that low concentrations of ROS are essential for sustaining redox signaling that maintains homeostasis and enables adaptation to metabolic and environmental stress [8].

However, when ROS generation surpasses the buffering capacity of antioxidant systems, a state of oxidative stress arises [9]. This imbalance leads to oxidation of lipids, proteins, and nucleic acids, with functional impairment and structural damage as consequences [10,11]. Oxidative DNA lesions such as 8-oxo-2′-deoxyguanosine exemplify the mutagenic potential of chronic ROS exposure [12]. As a result, prolonged oxidative stress can contribute to the development of a wide range of pathological conditions, including cancer, cardiovascular disease, metabolic disorders, neurodegenerative diseases, and infertility [13,14,15,16,17]. In this context, aging is increasingly recognized as a condition characterized by cumulative oxidative damage and progressive mitochondrial dysfunction, which are associated with age-related functional decline and disease susceptibility [18,19].

To counteract ROS, cells employ an elaborate antioxidant defense network. Antioxidant enzymes, such as superoxide dismutase (SOD), catalase (CAT), glutathione peroxidase (GPx), and peroxiredoxins work together with small antioxidant molecules like glutathione, vitamins C and E, carotenoids, and ferritin to protect cells from oxidative damage [20]. These antioxidant systems are spatially compartmentalized within distinct cellular regions, enabling localized control of ROS levels while preventing uncontrolled propagation of oxidative damage. These systems not only detoxify ROS but also regulate redox signaling by modulating reversible oxidative modifications of protein thiols [20,21]. In this sense, ROS occupy a dual role: essential for signaling and host defense at physiological levels, but drivers of molecular damage and disease when produced in excess [22]. This duality highlights why oxidative stress is often regarded as a central contributing mechanism linking environmental exposures, cellular metabolism, and chronic disease development.

### 1.2. Unavoidable Oxidative Stress in Space: The Critical Role of Diet and the Microbiome

**Figure 1:**
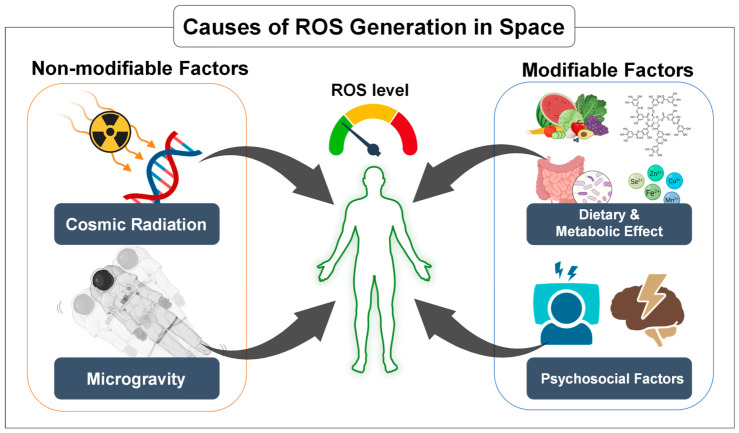
Major contributors to reactive oxygen species (ROS) generation during spaceflight. Astronauts are exposed to multiple stressors that increase ROS production and disrupt redox homeostasis. Key contributors include cosmic radiation and microgravity, which promote DNA damage and mitochondrial dysfunction, as well as dietary and metabolic factors and psychosocial stressors, such as altered nutrition, microbiome changes, confinement, and circadian disruption. Together, these factors interact to elevate oxidative stress during long-duration space missions. Created in BioRender. Song, M.S. (2026) https://BioRender.com/abvcq34, accessed on 16 April 2026.

Astronauts are exposed to a space environment that promotes increased ROS production and consequently elevates the risk of oxidative stress. Among the different factors present in space, exposure to ionizing radiation is considered one of the most significant contributors to oxidative stress [23]. Galactic cosmic rays and solar particle events penetrate spacecraft shielding and interact with biological tissues to generate free radicals, initiating cascades of lipid peroxidation, protein oxidation, and DNA damage [24,25,26]. It has been reported that radiation is one of the major factors driving oxidative stress and damage, affecting bone, cardiovascular, immune, and nervous systems [27,28,29,30]. Microgravity represents another major driver of oxidative imbalance, as it disrupts cellular homeostasis, alters mitochondrial function, and enhances ROS production, thereby contributing to sustained oxidative stress in spaceflight conditions [31]. At the mitochondrial level, both ionizing radiation and microgravity have been shown to perturb electron transport chain function, particularly at complexes I and III, increasing electron leakage and superoxide generation. In addition to microgravity and radiation, other stressors inherent to spaceflight further contribute to redox imbalance [32,33]. Deep-space hypomagnetic fields (HMF) have been reported to disrupt cellular homeostasis, partly through modulation of ROS production. For instance, mouse skeletal muscle cells exposed to the HMF showed higher ROS levels and reduced cell viability, which was attributed to the reduction in mitochondrial activity and of the energy production [34]. Similar mitochondrial dysfunction and ROS accumulation have been observed in hippocampal cells, with effects intensifying alongside the duration of exposure [35]. Beyond these cellular impacts, long-term HMF exposure alters the gut microbiota composition, notably increasing the Firmicutes to Bacteroidetes ratio (F/B) ratio and reducing fecal short-chain fatty acid (SCFA) concentrations [36]. These shifts, along with HMF-induced reproductive and circadian disruptions [37,38], may contribute to systemic redox imbalance and potentially interact with other spaceflight stressors, including radiation and microgravity. Furthermore, non-environmental stressors such as disruption of circadian rhythms, confinement, and psychosocial stress activate neuroendocrine pathways that further modulate mitochondrial function and ROS production [39,40,41]. Evidence from astronaut cohorts and ground-based analogs indicates that these combined challenges elicit systemic oxidative stress responses across multiple organ systems, including the immune and cardiovascular systems [31,42] (Figure 1).

Importantly, both diet and the gut microbiota should be recognized as critical determinants of oxidative balance in space. Diet is relevant not only for its impact on microbial ecology but also as an independent factor. The nutrient composition of space food, the preservation and processing methods required for long-term storage, and the altered eating patterns that occur during missions can directly influence oxidative stress [43,44]. At the same time, the gut microbiota strongly shapes host redox homeostasis, and its composition may shift not only in response to diet but also under other spaceflight stressors, such as radiation and microgravity [45,46,47]. Conversely, when diet is well managed and microbial stability is maintained, these factors can enhance antioxidant defenses, support mitochondrial function, and help sustain systemic resilience. Accordingly, diet and gut microbiota should not be viewed as secondary contributors but as central regulators of oxidative balance in space. These factors can either exacerbate or mitigate oxidative stress depending on their composition and stability. Therefore, they should be considered in an integrated manner rather than as independent variables, as their interaction plays a key role in maintaining systemic redox homeostasis.

In the difficult environment of space, astronauts face four main causes of harmful oxidative stress, including microgravity, radiation, mental stress, and diet. Among those, diet and eating habits are the easiest things to change. Unlike radiation or gravity, which we cannot avoid, we can choose and plan our food carefully [48,49]. Choosing the right food not only lowers stress from the diet itself but also strengthens the body against other stressors in space. In this review, we aim to integrate current evidence on how diet and the gut microbiome, both individually and in combination, influence redox homeostasis in space, and to discuss how these insights may inform nutritional and microbial strategies to protect astronaut health during long-duration missions.

## 2. Space Food Systems and Oxidative Stress

Long-duration space missions rely heavily on shelf-stable foods that can be stored for extended periods without refrigeration. Although these foods are designed to ensure microbial safety and simplify mission logistics, their nutrient composition can change during storage, processing, and packaging. Such changes may influence systemic oxidative stress in astronauts (Table 1), which summarizes the key dietary and microbiome-related factors contributing to oxidative stress during spaceflight.

**Table 1:** Dietary and food-system factors contributing to oxidative stress during spaceflight and potential mitigation strategies.

	Source of Oxidative Stress	Mechanism	Reference
Nutritional Quality and Space food	Nutrient degradation	Loss of antioxidants (Vitamin C, A, B1, and folate)	[50,51,52,53]
	Food processing	Heat-induced Maillard reactions	[54]
		Irradiation-induced lipid peroxidation	[53,55,56]
	Packaging	Bisphenol A	[57,58,59,60]
		Phthalates	[61,62,63,64]
		Aluminum	[65,66,67,68]
	Diet composition	Constraints of energy needs, appetite, and logistics	[48,49,51,69]
		High-fat intake	[70,71,72,73]
		High-protein intake	[74,75,76,77]
Spaceflight dietary habit	Microgravity	Alterations in gastrointestinal motility	[78,79,80]
		Impaired intestinal barrier integrity	[81,82,83]
	Dehydration	Dehydration by body fluid redistribution	[49,84,85]
		Limited food moisture due to upmass limitation	[49,53,86,87]
		Reduced water intake	[49,85,88]
	Psychological factor	Reduced food intake	[69,89,90,91]
		Reduced intake of antioxidant	[50,51,52,89]
		Psychobiological stressors	[39,40,41,92]
	Eating behavior	Altered meal timing	[93,94,95]
		Space motion sickness	[79,96,97]
		Circadian rhythm	[39,94,98]
Microbiome	Alterations in microbiota	Functional properties	[99,100,101,102]
		Gut microbial diversity	[46,101,102,103]
		Firmicutes/Bacteroidetes ratio	[36,46,101,103]
		Lactobacillus	[46,101,104,105]

### 2.1. Nutrient Degradation and Antioxidant Loss

Space food systems are subject to significant degradation of essential vitamins and antioxidants during long-duration missions. For example, vitamin C levels can decrease substantially within the first year, in some cases approaching near-complete loss, with substantial losses also noted for vitamins A, thiamine, and folate [50,51]. This decline in antioxidants has important redox-related health implications and is associated with increased susceptibility to oxidative stress.

Vitamin C plays a crucial role in scavenging ROS and regenerating vitamin E. When these antioxidants are depleted, the body’s defenses against lipid peroxidation are weakened, leading to the accumulation of harmful compounds, such as H_2_O_2_ and O_2_^•−^ and the formation of toxic byproducts, including malondialdehyde (MDA) and 4-hydroxynonenal (4-HNE). This effect has been observed in astronauts: after 4–6 months on the International Space Station (ISS), plasma antioxidant concentrations decreased while urinary 8-OHdG, a marker of oxidative DNA damage, increased [89].

Given that space foods are designed to remain stable for three to five years, cumulative nutrient loss may place crew members at risk for undernutrition [52]. This is particularly concerning because vitamins C, E, and A function as major dietary antioxidants, and their depletion increases susceptibility to oxidative stress. Earth-based studies further demonstrate that prolonged storage and exposure to radiation can accelerate oxidation of dietary fatty acids and degradation of vitamins [50,53]. These processes not only reduce nutrient effectiveness but also generate oxidative byproducts. Consequently, the space food system may provide insufficient antioxidant support, potentially increasing susceptibility to oxidative stress in astronauts.

### 2.2. Processing- and Storage-Induced Oxidative Reactions

To ensure food safety and long-term shelf stability, space foods undergo processing methods like thermal sterilization and irradiation. While these treatments effectively eliminate microbial contaminants, they also promote oxidative reactions within the food matrix. Heat treatment induces Maillard reactions, which generate advanced glycation end products (AGEs). These AGEs have been reported to contribute to increased intracellular ROS production upon consumption [54]. Similarly, irradiation accelerates lipid peroxidation. For instance, gamma-irradiated chicken meat and meat products exhibit significantly elevated thiobarbituric acid reactive substances (TBARS), a robust marker of lipid oxidation [55]. Specifically, the meta-analysis showed that gamma irradiation consistently increased TBARS levels across multiple storage intervals (days 0, 7, and 14), confirming enhanced oxidative degradation of PUFAs. Moreover, recent experimental work demonstrated that gamma irradiation applied to smoked chicken accelerates lipid oxidation and leads to the formation of aldehydic volatile compounds—including notable increases in MDA-related markers [56]. Over long storage periods, these oxidative byproducts accumulate as nutrient levels decline [52]. Antioxidant vitamins—including A, C, thiamine, and folate—are particularly vulnerable to degradation from thermal stress and subsequent oxidation. Furthermore, the absence of refrigeration or inert-atmosphere packaging on current space missions means foods are stored at ambient temperatures with some oxygen present, facilitating ongoing oxidative deterioration. Fat-rich foods, especially those with unsaturated fats, are susceptible to rancidity and demonstrate limited stability over an extended shelf life. Ultimately, these oxidative processes diminish the nutritional quality of space food and may contribute to increased oxidative burden upon consumption, particularly under conditions of limited antioxidant intake.

### 2.3. Packaging-Related Pro-Oxidant Exposure

For long-duration space missions, multilayer polymer/foil packaging is indispensable for safety and shelf-life, but some materials can leach small molecules into foods and thereby add pro-oxidant burden to the astronauts. Recent migration studies show that bisphenol A (BPA) can be released from epoxy can linings into foods and food simulants under ordinary conditions (e.g., aqueous and lipid media), directly documenting BPA transfer from packaging to food matrices [57]. Likewise, phthalate plasticizers migrate from plastic containers into foods and food simulants during storage/heating; controlled experiments quantifying dibutyl phthalate (DBP), benzyl butyl phthalate (BBP), and other plasticizers show measurable migration that significantly increases at higher temperatures [106], and surveys of convenience foods detect multiple phthalates at quantifiable levels [61]. Once in the body, BPA exerts oxidative stress via mitochondrial dysfunction in target organs. In animal models, dietary exposure to BPA reduces oxygen consumption, ATP production, and mitochondrial membrane potential while increasing hepatic oxidative stress markers—providing evidence that BPA can disrupt mitochondrial bioenergetics [58]. In vivo work in rats further shows renal mitochondrial impairment and oxidative damage after BPA exposure, reinforcing a mechanistic link to organ toxicity [107]. Phthalates are lipophilic and concentrate preferentially in fatty foods, which is operationally important for many energy-dense space items. Analytical studies report higher DBP/DEHP levels in vegetable and olive oils compared with hydrophilic foods, and measured migration into oils can reach the µg–mg kg^−1^ range depending on matrix and conditions [62,108]. These exposures matter physiologically because several phthalates can drive oxidative and inflammatory signaling downstream of mitochondrial or NADPH-oxidase activation.

Aluminum-based packaging introduces a separate pro-oxidant pathway. Recent migration studies show substantial aluminum dissolution into acidic foods (e.g., tomato sauces/purées) during normal cooking/holding in uncoated aluminum pans or trays; aluminum levels in tomato products can reach approximately 80–130 mg Al/kg, and release significantly increases as pH drops and temperature rises [65]. Recent quantifications under household-like baking/foil use confirm measurable Al leaching into meats and fish, modulated by seasoning/marinade acidity [66]. Importantly, dietary aluminum can provoke brain oxidative stress in vivo: rodent studies have reported increased lipid peroxidation and reduced antioxidant enzyme activities following aluminum exposure, consistent with elevated oxidative stress [109,110].

While advanced packaging is essential for safety and logistics, migrants like BPA and phthalates (from polymers) and Al (from foil/containers) can (i) enter packaged foods eaten frequently on mission and (ii) act as pro-oxidants by impairing mitochondrial function or amplifying lipid peroxidation. In diets already constrained in fresh antioxidants, these additional exogenous ROS drivers may further contribute to the overall oxidative burden under spaceflight conditions.

### 2.4. Macronutrient Composition and Metabolic ROS Production

Because mass and volume constraints favor energy-dense menus, spaceflight diets often skew toward higher proportions of fat and high-quality protein. While operationally practical, this macronutrient pattern can increase overall oxidative burden and interact adversely with microgravity-induced mitochondrial vulnerability. High-fat intake has been associated with increased mitochondrial electron flux and ROS generation [70], which may further exacerbate oxidative stress under spaceflight-induced mitochondrial stress conditions [71,111]. Similarly, high protein intake has been associated with increased ROS generation through xanthine oxidoreductase (XO/XOR) activity [74]. In addition, diet-induced acid load may contribute to oxidative signaling and bone resorption under spaceflight conditions [75,76].

Compounding these mechanistic links, mission menus commonly target ~55% carbohydrate, ~30% fat, and ~15% protein—values aligned with NASA’s operational nutrition. However, meeting energy goals under appetite and logistics constraints can still push crews toward disproportionately fatty or protein-heavy choices with insufficient antioxidant-rich produce. This combination may increase metabolic ROS and reduce antioxidant intake [112]. In sum, the macronutrient profile of space food, if overweighted toward fat and animal protein and combined with limited intake of fruits and vegetables, can amplify mitochondrial ROS generation (fat-driven) and XO-derived oxidants (protein/purine-driven) under spaceflight-induced mitochondrial stress, unless balanced by dietary buffering (K-rich plants), omega-3s, and antioxidant intake [111].

## 3. Dietary Behaviors and Physiological Changes During Spaceflight

Beyond the composition of space food itself, the way astronauts consume these foods in microgravity and the physiological alterations induced by spaceflight significantly influence oxidative stress. During spaceflight, the human body undergoes physiological changes affecting digestive function, hydration, appetite, and eating behavior. If these factors are not adequately managed, they may exacerbate redox imbalance.

### 3.1. Gastrointestinal Function in Microgravity

The fundamental alteration of gastrointestinal (GI) physiology in microgravity arises from the reliance of digestion primarily on peristaltic contractions in the absence of gravitational assistance. Evidence from human analog studies indicates that GI transit may become altered under microgravity conditions, although reported outcomes vary depending on experimental context. For example, dry immersion studies showed no significant change in gastric emptying of liquid food but revealed region-specific alterations in intestinal transit, including delayed large intestinal evacuation [78,113]. In contrast, head-down tilt bed rest models have reported enhanced intestinal motility without overt GI symptoms [79], underscoring the context-dependent effects of microgravity on GI function.

At the cellular level, simulated microgravity disrupts the coordinated regulation of GI motility by affecting interstitial cells of Cajal and GI hormone secretion, leading to impaired rhythmic contractions and inefficient nutrient absorption [80].

Beyond motility, microgravity markedly impairs intestinal barrier integrity. Experimental evidence from both rodent tail-suspension models and human intestinal epithelial cell cultures demonstrates that simulated weightlessness disrupts apical junction localization and significantly reduces the expression of tight junction proteins, including occludin and ZO-1 [81,82]. Consistently, a 21-day unloading study reported decreased levels of occludin, claudin-1, claudin-5, and E-cadherin in the ileum, accompanied by activation of TLR4/MyD88/NF-κB signaling pathway [83]. In vitro, intestinal epithelial cells cultured under simulated microgravity conditions exhibit delayed tight junction assembly, reduced transepithelial electrical resistance, and increased paracellular permeability [82]. Collectively, these changes promote barrier dysfunction, facilitating endotoxin translocation and triggering inflammatory signaling and systemic ROS production.

Taken together, microgravity-induced alterations in GI motility and epithelial barrier function converge to impair digestion and nutrient absorption, while simultaneously amplifying oxidative stress. Consequently, the GI tract emerges not only as a compromised digestive system under spaceflight conditions but also as a potential contributor to systemic ROS burden.

### 3.2. Hydration Constraints

Adequate water intake is fundamental for physiological processes, such as circulation, metabolism, thermoregulation, and nutrient transport. In microgravity, body fluids redistribute toward the head, leading to reduced plasma volume and blunted thirst perception. Studies simulating short-duration spaceflight report an approximately 10% reduction in plasma volume within the first days, increasing the risk of fluid imbalance and underhydration [84]. In addition, the use of pouches or straws for liquid consumption alters drinking behavior and may further limit total fluid intake.

Dehydration has been associated with oxidative stress on Earth. Recent human studies demonstrate that fluid deficits elevate systemic and intramuscular oxidative stress markers (e.g., H_2_O_2_) and alter cellular growth signaling, indicating that even modest fluid deficits increase ROS burden [85]. Accordingly, astronauts already exposed to radiation- and microgravity-related oxidative stress may experience additive redox strain when hydration is insufficient.

Due to limited water resources, space foods are largely dehydrated or freeze-dried and rehydrated on orbit with strictly rationed water supplies. As a result, foods are formulated with defined moisture levels, and NASA’s food system relies on intermediate-moisture items that ensure safety and palatability without excessive water use [86,87]. Inadequate hydration or suboptimal food moisture can impair swallowing and digestion, whereas balanced textures facilitate safe consumption in microgravity.

These hydration constraints have direct implications for oxidative stress. Reduced water intake can increase renal and metabolic strain—conditions known to elevate ROS production in terrestrial studies—and may compromise antioxidant enzyme activity that depends on stable aqueous and electrolyte environments [88]. Thus, although water rationing and low-moisture food formulations are operationally essential, they may unintentionally exacerbate oxidative imbalance during long-duration missions.

### 3.3. Menu Fatigue and Underconsumption

Extended residence in confined habitats severely limits dietary variety, requiring crews to repeatedly consume a finite set of pre-packaged foods. Operational records and flight physiology studies consistently show that astronauts consume less energy than required, with a mean in-flight intake of approximately 80% of energy needs on ISS missions. This underconsumption is accompanied by biochemical signs of oxidative damage, including the fact that insufficient intake weakens antioxidant defenses and heightens redox stress during long-duration missions [89].

Menu monotony and reduced palatability are frequently cited as drivers of appetite decline, and group-based head-down bed rest (HDBR) studies recapitulate this pattern. In controlled HDBR experiments, spontaneous reductions in ad libitum intake of ~12–17% have been reported over several weeks, demonstrating that unloading and environmental constraints alone can induce reproducible hypophagia [90,91]. Endocrine measurements during unloading further support this mechanism, with alterations in leptin and ghrelin signaling consistent with disrupted appetite regulation under simulated microgravity and circadian stress [114].

Underconsumption has direct redox consequences because it reduces the intake of antioxidant micronutrients, including vitamins C and E, carotenoids, selenium, and polyphenols, while spaceflight-associated mitochondrial stress persists. ISS cohort data and bed-rest analogs reveal concurrent shifts in oxidative stress markers, iron indices, and micronutrient handling. Recent clinical trials (e.g., AGBRESA) further indicate that simulated microgravity can sustainably increase serum iron availability and hepcidin levels, particularly in males, which may elevate the risk of iron-induced oxidative damage over mission timescales [89,115].

Psychobiological stressors, such as confinement, sleep and circadian disruption, and elevated thermal load further compound appetite suppression and inflammatory tone during spaceflight. For example, long-duration ISS missions are associated with progressive increases in core body temperature, an inflammatory state that can suppress appetite and exacerbate oxidative burden, reinforcing the risk of persistent energy and antioxidant deficits in monotonous food environments [92].

Importantly, menu fatigue–induced undernutrition should not be viewed solely as a behavioral phenomenon but rather as a multifactorial physiological and operational issue. In constrained food systems with limited variety, repeated exposure to identical meals progressively reduces palatability, leading to selective avoidance and declining intake over time [43]. This process is further exacerbated by spaceflight-specific factors, including altered sensory perception, early-flight anorexia associated with space motion sickness, and increased workload or time constraints that limit structured eating opportunities [116]. In addition, prolonged storage and processing-related changes in texture and flavor can further diminish food acceptability, reinforcing the cycle of reduced intake [117].

From an operational perspective, early detection of underconsumption is critical. Continuous monitoring of food intake patterns, acceptability ratings, and body mass changes can provide early-warning indicators of developing nutritional deficits [48,76]. Multi-attribute assessments of food acceptability—including taste, texture, aroma, and overall satisfaction—combined with tracking of dietary diversity and frequency of consumption, may enable timely identification of menu fatigue before it translates into clinically relevant undernutrition [69].

To mitigate menu fatigue–associated oxidative risk, integrated strategies are required at both the food system and behavioral levels. Increasing menu diversity and enabling personalized food selection within logistical constraints can reduce repetitive exposure and improve long-term adherence. Sensory optimization and operational strategies, including protected mealtime scheduling and reduced preparation burden, are also essential [48,69,116].

Taken together, menu fatigue–induced underconsumption represents an important driver of oxidative imbalance during spaceflight. Beyond simple caloric deficiency, it reflects a complex interaction between behavioral, physiological, and operational factors that collectively reduce antioxidant intake while amplifying metabolic and mitochondrial stress. Without effective monitoring and mitigation, this sustained imbalance may weaken endogenous antioxidant defenses and increase susceptibility to ROS-mediated damage during long-duration missions.

### 3.4. Eating Behavior and Circadian Disruption

Microgravity and the spaceflight environment alter not only what astronauts eat but also how and when food is consumed. Beyond total caloric intake, changes in eating behavior, meal timing, and the physiological context of food consumption represent additional pathways through which spaceflight may influence metabolic regulation and oxidative stress.

Altered nutrient handling has been observed under spaceflight conditions. Metabolic profiling of astronauts aboard the ISS indicates a postprandial shift in substrate utilization toward carbohydrates over lipids [93]. Such changes may modify mitochondrial electron flux and antioxidant demand, providing a plausible link between altered meal timing, nutrient processing, and redox homeostasis.

Behavioral and physiological factors further shape eating patterns in microgravity. Space motion sickness (SMS), affecting approximately 60–80% of astronauts during the early phase of flight, commonly induces nausea and transient appetite suppression, encouraging smaller and more frequent meals [96,97]. Even after adaptation, altered gas–liquid dynamics in microgravity continue to influence meal size and distribution as crews adjust eating strategies to minimize gastrointestinal discomfort.

Circadian rhythm disruption constitutes an additional and distinct contributor. Misalignment of sleep–wake cycles and exposure to artificial lighting have been associated with increased oxidative stress markers and altered cortisol rhythms [98], as well as impaired antioxidant defenses and metabolic dysregulation [94]. In the context of spaceflight, such circadian disturbances may destabilize meal timing and hormonal regulation of metabolism, indirectly shaping redox balance.

Taken together, alterations in eating behavior, meal timing, metabolic processing, and circadian regulation—rather than caloric intake alone—represent additional pathways through which spaceflight may influence oxidative stress. These considerations support the integration of chrononutrition, behavioral scheduling, and circadian alignment into strategies aimed at mitigating redox imbalance during long-duration missions [95].

## 4. Changes in the Astronaut Gut During Spaceflight

Quantitative evaluations suggest that an average adult harbors approximately 3.0 × 10^13^ human cells and about 3.8 × 10^13^ bacterial cells, implying a near 1.3:1 bacterial-to-human cell ratio [118]. Variability between individuals due to differences in body composition or physiological factors may affect this ratio [119]. Comprehensive reviews further indicate that the human body contains on the order of 10^13^ to 10^14^ bacterial cells, underscoring the microbiome as a major contributor to host physiology [120]. Within the gut, the microbial community comprises hundreds to thousands of species and collectively encodes a vast metagenome that contains millions of unique genes, far exceeding the genetic repertoire of the human genome [121]. Microbial diversity is observed across multiple body sites, including the skin, where a single hand can harbor over 150 distinct species-level phylotypes with limited overlap even between individuals [122,123]. However, in the context of spaceflight-induced metabolic and redox alterations, the gut microbiome represents the most relevant and extensively studied compartment.

Astronauts encounter multiple stress factors during spaceflight that can disrupt the balance of their microbiota. Research carried out in both actual space missions and ground-based microgravity simulations indicates that spaceflight conditions can alter the functional properties of commensal and opportunistic bacteria. These alterations may include shifts in virulence, enhanced biofilm formation, modified growth behavior, and changes in antibiotic resistance profiles [99,100,101,124]. Although results concerning microbiome diversity at different body sites remain inconsistent, with some studies describing either increases or decreases in skin, nasal, or oral communities, there is general agreement that gut microbial diversity exhibits significant alterations during spaceflight [101,103]. These alterations, although variable in direction depending on mission duration and individual physiology, are consistently observed and highlight the gut as a particularly sensitive microbial niche in the space environment. Both mouse models flown on the ISS and longitudinal studies of astronauts indicate that spaceflight is associated with distinct shifts in the gut microbiome [46,103]. These include an overall increase in *Firmicutes* (notably *Clostridiales* such as *Faecalibacterium*), coupled with a decrease in typically beneficial taxa such as *Lactobacillales* and *Akkermansia* [46,101]. While some SCFA-producing bacteria expanded, the marked reduction in anti-inflammatory genera like *Akkermansia* has been linked to heightened inflammatory responses during space missions [103]. Historical observations from the Skylab program further complement these findings, showing a reduction in gastrointestinal diversity despite an overall rise in microbial load, with pathogenic strains such as *Serratia marcescens* and *Staphylococcus aureus* increasing in relative abundance, the latter even being transmitted among crew members [125]. Together, these findings indicate that dysbiosis and increased pathogen transmission are key features of the spaceflight-associated microbiome, highlighting the need for targeted microbial and dietary countermeasures.

## 5. Microbiome Shifts as Drivers of ROS Generation and Oxidative Stress in Space

Although findings are not uniform across all studies, evidence indicates that spaceflight can influence the F/B ratio, with several investigations in both rodent models and astronauts reporting an increase during missions [46,101,102,103,126]. In mice flown on the ISS, the F/B ratio increased significantly relative to ground controls [46]. Consistent with these findings, Liu et al. analyzed fecal samples from astronauts during the Shenzhou-11 mission and reported that *Firmicutes* abundance gradually increased while *Bacteroidetes* decreased after spaceflight, indicating a similar rise in the F/B ratio at the human level [101]. More recently, Bedree et al. examined rodents from the Rodent Research-5 mission and showed that spaceflight not only led to bone loss but was also accompanied by gut microbiome alterations, including an increased F/B ratio [126]. Together, these studies from both rodent and human models collectively indicate that spaceflight is frequently associated with an increased F/B ratio. On Earth, similar microbial shifts have been reported in metabolic syndrome, obesity, and aging, conditions where oxidative stress is a defining feature [127,128,129]. Supporting this connection, a human cohort study demonstrated a positive correlation between the F/B ratio and systemic oxidative stress status, as reflected by the oxidative stress index (OSI) in pregnant women [130]. Similarly, in an animal toxicology model, exposure to aflatoxin B1 significantly increased the F/B ratio while simultaneously elevating oxidative stress markers, including reduced antioxidant enzyme activities and higher lipid peroxidation [131]. In addition, several spaceflight studies have reported reductions in *Lactobacillus* and related *Lactobacillales*, although such findings are not uniform. Jiang et al. and Liu et al. both reported post-flight declines in *Lactobacillus* abundance [46,101]. On Earth, *Lactobacillus* species are well known for their probiotic and antioxidant roles [104]. For example, administration of *Lactobacillus* plantarum in a murine oxidative stress model increased SOD, CAT, and GPx while reducing MDA [105].

The traditional view of spaceflight-induced dysbiosis is evolving toward a more dynamic understanding of the microbiome-redox axis. Recent longitudinal data from the Space Omics and Medical Atlas (SOMA), particularly the 3-day Inspiration4 mission, have provided a pivotal observation that the human microbiome undergoes rapid and significant re-patterning even within an ultra-short-duration window. A notable finding from this cohort was the homogenization effect, where the microbial distributions among crew members became increasingly similar, reflecting the powerful selective pressure of the spacecraft environment and shared stressors [132,133,134]. Crucially, these early-phase microbial shifts coincided with systemic physiological changes. Data from the Inspiration4 crew revealed immediate post-flight elevations in pro-inflammatory cytokines, including IL-6 and CCL2, alongside a significant rise in malondialdehyde (MDA), a key biomarker of lipid peroxidation [42,132]. Moreover, High-resolution multi-omic profiling from the Inspiration4 mission has revealed that spaceflight-induced immune dysregulation is deeply intertwined with microbiome shifts [135]. This confirms that the microbiome is not merely a bystander but a central immune-metabolic hub that dictates host physiological adaptation to the space environment. Although the specific directional changes in the F/B ratio or the abundance of *Lactobacillus* species may vary across different pilot studies and individual astronaut responses, the temporal synchronicity between microbial instability and oxidative distress is undeniable.

Taken together, these findings suggest that spaceflight-related alterations in the F/B ratio and *Lactobacillales* levels may increase susceptibility to oxidative stress by compromising host antioxidant defenses. However, the causal role of these microbial changes in astronaut health remains to be established and requires further validation through dedicated spaceflight studies.

## 6. Toward an Integrated Redox-Management Strategy for Spaceflight

Spaceflight-induced oxidative stress arises from the convergence of multiple, interdependent drivers, including ionizing radiation, microgravity-associated mitochondrial dysfunction, dietary constraints, microbiome alterations, and behavioral or circadian stressors [25,31,42,43,47,93,94,136,137]. As discussed throughout this review, these factors rarely operate as isolated inputs; rather, they interact over time to amplify redox imbalance across tissues and physiological systems, thereby increasing susceptibility to oxidative distress during long-duration missions [42,111]. Consequently, effective mitigation cannot rely on single countermeasures or static, one-size-fits-all prescriptions. Future strategies must instead adopt an integrated framework that treats oxidative stress as a systems-level challenge shaped by mission phase, individual biology, and coupled host–environment interactions [33,111,138].

Within this framework, nutrition should be positioned as both a primary protective layer and a potential source of avoidable oxidative burden. Because antioxidant micronutrients decline during prolonged ambient storage in the space food system, preserving labile vitamins and minimizing oxidative degradation through improved processing and high-barrier packaging is a first-line defense [50,52,86,87,89]. In parallel, macronutrient composition should be treated as a modifiable determinant of endogenous ROS generation. Diets skewed toward higher fat and/or excessive animal protein can shift mitochondrial electron flux and purine-derived oxidant production, while increased dietary acid load can exacerbate downstream physiological stress relevant to spaceflight (e.g., bone turnover) [72,73,75,76,77,111]. Equally important is the reduction in diet-derived pro-oxidant exposures, including food-contact migrants, such as bisphenols, phthalates, and reactive metals, which may add mitochondrial and inflammatory burden in settings already constrained in fresh antioxidant intake [59,60,61,63,64,66,67,68,107,139,140]. Together, these considerations argue that “space nutrition” should be framed not merely as meeting caloric targets, but as engineering a diet optimized for redox stability and resilience.

At the same time, the gut microbiome should be recognized as a dynamic regulator of host redox homeostasis and inflammatory tone. Spaceflight and space-relevant stressors can alter microbial composition and function, potentially weakening barrier integrity and promoting pro-inflammatory signaling that amplifies systemic ROS production [45,46,47]. Conversely, microbiome-supportive dietary strategies provide a promising route to reinforce host resilience under spaceflight conditions.

**Figure 2:**
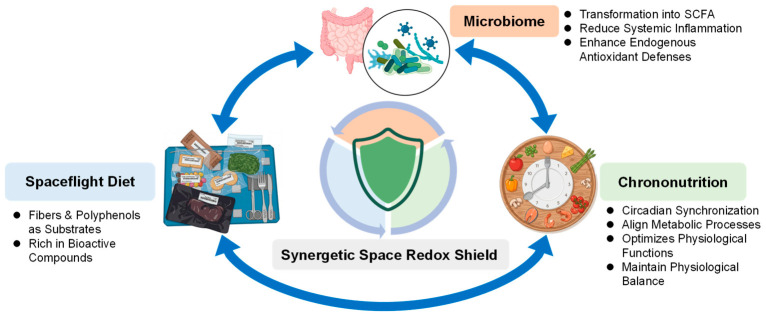
Schematic of the Synergetic Space Redox Shield. This figure illustrates the integrated strategy for managing oxidative stress during space exploration through the functional interactions between three key pillars. Dietary fibers and polyphenols act as essential substrates for gut microbes, which transform them into bioactive metabolites (such as SCFAs) that strengthen the body’s internal antioxidant defenses and reduce inflammation. Chrononutrition (meal timing) acts as a circadian synchronizer, aligning these metabolic processes with the body’s internal clock. The blue arrows represent the crosstalk between these interventions, highlighting how their combined effect creates a synergy that maintains a robust redox balance and protects astronaut health during long-duration missions. Created in BioRender. Song, M.S. (2026) https://BioRender.com/0ep9p8s, accessed on 16 April 2026.

Microbiome-supportive diets incorporating fiber, resistant starches, and natural compounds such as polyphenols and flavonoids provide a plausible strategy to support intestinal barrier function and redox homeostasis. These dietary components can act through dual mechanisms, including direct antioxidant effects and modulation of the gut microbiome. In particular, fibers and polyphenols serve as key substrates for beneficial microbial taxa, promoting the production of bioactive metabolites such as short-chain fatty acids (SCFAs), which are associated with reduced inflammation, enhanced endogenous antioxidant defenses, and improved redox balance [43,47,102,141]. In addition, the functional effects of these dietary interventions are closely linked to metabolic timing, as scheduled meal intake can act as a circadian synchronizer aligning microbial and host physiology [142,143]. These interactions support a coordinated framework in which dietary composition, microbiome activity, and chrononutrition collectively contribute to maintaining physiological stability during long-duration missions (Figure 2).

Targeted antioxidant supplementation represents an additional—but secondary—layer of defense that should be deployed with mechanistic and operational discipline. While antioxidant cocktails and supplements have been proposed as countermeasures against ROS-mediated cellular injury during crewed missions, a recent meta-analysis underscores that efficacy is highly dependent on compound selection, dosing, and timing-with pre-radiation administration showing the most significant protective effects against radiation-induced DNA damage [144,145]. Accordingly, antioxidant strategies should be pathway-aware, temporally controlled (e.g., mission-phase- or risk-window-guided), and individualized.

This need for individualized intervention is further supported by multi-omics and longitudinal profiling efforts in astronauts and spaceflight-related cohorts, which highlight substantial inter-individual variability in baseline redox capacity and stress responses [33,111,138]. In addition to these sex-specific findings, general factors such as age-related reductions in mitochondrial bioenergetic reserves and the pre-flight abundance of antioxidant-supportive taxa in the baseline microbiome further define an individual’s redox resilience profile [19,130,131,146]. Integrating these stratifying factors into a precision redox-management framework is therefore essential for tailoring dietary and microbial interventions to the specific biological needs of diverse crew members.

Among these determinants, sex has emerged as a critical biological factor influencing redox responses to spaceflight. Specifically, recent high-resolution data from the SOMA and the Inspiration4 mission have elucidated distinct sex-specific trajectories in biological responses, with male astronauts showing more pronounced gene expression changes and slower recovery of chromatin accessibility compared with females [135].

To effectively tailor these interventions, the development of precision space nutrition must account for physiological differences between sexes [147,148]. Estrogen, for example, plays a protective role in cardiovascular regulation, bone remodeling, and antioxidant defenses. Recent evidence highlights that spaceflight-induced oxidative stress is a central driver of skeletal deterioration, with particular vulnerabilities linked to estrogen deficiency in female astronauts [149,150]. Additionally, male and female astronauts exhibit distinct endocrine and metabolic adaptations. Males tend to show greater increases in spaceflight-induced insulin resistance, whereas females experience more pronounced changes in renin-aldosterone regulation, along with slower metabolic recovery timelines post-flight [147,148]. Accordingly, practical dietary formulations should be adjusted to reflect these differences—as outlined in Table 2—by fine-tuning antioxidant intake (e.g., phytoestrogens for females), macronutrient composition, and micronutrients such as iron [49], in order to optimally support both male and female astronauts.

**Figure 3:**
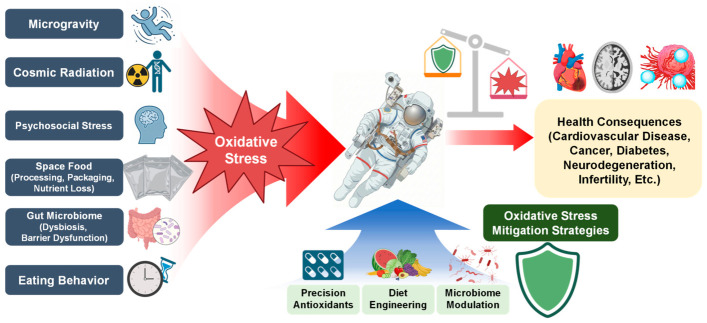
Sources of oxidative stress during spaceflight and potential mitigation strategies. Multiple factors in the space environment—including microgravity, cosmic radiation, psychosocial stress, characteristics of space food (processing, packaging, and nutrient loss), alterations in the gut microbiome, and changes in eating behavior—can increase ROS production and disrupt redox homeostasis. These processes may contribute to adverse health outcomes, such as cardiovascular disease, cancer, diabetes, neurodegeneration, and infertility. Potential mitigation strategies include precision antioxidant supplementation, dietary engineering to improve nutritional quality, and microbiome-targeted interventions. Created in BioRender. Song, M.S. (2026) https://BioRender.com/abvcq34, accessed on 16 April 2026.

Taken together, these personalized and sex-specific strategies highlight the need for a coordinated, system-level approach to redox management in spaceflight. Future redox countermeasures for long-duration spaceflight should be designed as an adaptive and interconnected system in which diet engineering, microbiome management, and antioxidant support are coordinated across mission phases and guided by biomarker-informed feedback [33,43,47,111,138] (Figure 3). The objective is not the elimination of ROS—which would be neither feasible nor desirable—but the maintenance of oxidative eustress under sustained spaceflight stressors [8,22]. Such an integrative redox-management approach provides a rational and biologically grounded roadmap for protecting astronaut health and performance during exploration-class missions beyond low Earth orbit.

## 7. Conclusions

Spaceflight exposes astronauts to a unique combination of environmental and physiological stressors that collectively elevate oxidative stress. Ionizing radiation, microgravity, dietary constraints, microbiome alterations, and behavioral factors interact in a dynamic and interdependent manner to disrupt redox homeostasis during long-duration missions.

This review highlights that, beyond unavoidable environmental challenges, modifiable factors—including space food system design, dietary composition, eating behaviors, and microbiome stability—play a central role in shaping oxidative balance in space. These factors act not in isolation but as interconnected drivers of redox disruption, while also providing multiple entry points for targeted intervention.

Future countermeasures should therefore move beyond isolated strategies and adopt integrated, system-level approaches that combine optimized space nutrition, microbiome-supportive diets, and targeted antioxidant management. Maintaining physiological redox balance—rather than eliminating ROS entirely—will be essential for protecting astronaut health and sustaining adaptive responses during long-duration space exploration missions.

## Figures and Tables

**Table 2:** Sex-specific physiological responses to spaceflight and tailored redox-nutritional strategies.

Physiological Metabolic Factors	Responses in Female Astronauts	Responses in Male Astronauts	Targeted Nutritional and Redox Strategy	References
Estrogen Signaling and ROS Defense	▪ Increased vulnerability to bone loss and cardiovascular shifts due to estrogen deficiency	▪ Lower reliance on estrogen-mediated endogenous antioxidant defenses	Phytoestrogens & Antioxidants: Increase polyphenols/flavonoids for females to buffer ROS and protect bone	[148,149]
	▪ Higher orthostatic intolerance			
Endocrine & Metabolic Adjustments	▪ Greater renin-aldosterone shifts	▪ Higher susceptibility to spaceflight-induced insulin resistance	Macronutrient Optimization: Adjust carbohydrate-to-fat ratios for insulin control (males) and metabolic recovery (females)	[147,150]
	▪ Prolonged post-flight metabolic and energy recovery	▪ Distance early-flight energy shifts		
Iron Storage and Metabolism	▪ Lower baseline iron stores	▪ Higher baseline serum ferritin	Precision Iron Management: Restrict iron and provide chelators (e.g., catechins) for males	[49,147]
	▪ Reduced risk of severe iron-overload toxicity	▪ Greater risk of iron-induced oxidative damage (via Fenton reaction)		

## Data Availability

No new data were created or analyzed in this study. Data sharing is not applicable to this article.

## Abbreviations

The following abbreviations are used in this manuscript:

ROS	Reactive oxygen species
SOD	Superoxide dismutase
CAT	Catalase
GPx	Glutathione peroxidase
MDA	Malondialdehyde
4-HNE	4-hydroxynonenal
AGEs	Advanced glycation end products
TBARS	Thiobarbituric acid reactive substances
BPA	Bisphenol A
DBP	Dibutyl phthalate
BBP	Benzyl butyl phthalate
HDBR	Head-down bed rest
SMS	Space motion sickness
OSI	Oxidative stress index
SCFA	Short-chain fatty acid
